# Towards Photocrosslinkable Lyotropic Blends of Organosolv Lignin and Hydroxypropyl Cellulose for 3D Printing by Direct Ink Writing

**DOI:** 10.3390/polym16202869

**Published:** 2024-10-10

**Authors:** Mehmet-Talha Yapa, Jacques Lalevée, Marie-Pierre Laborie

**Affiliations:** 1 Forest Biomaterials, Institute of Earth and Environmental Sciences, Faculty of Environment and Natural Resources, University of Freiburg, Werthmanstr. 6, 79085 Freiburg im Breisgau, Germany; mehmet.talha.yapa@biomat.uni-freiburg.de; 2 Freiburg Materials Research Center, Stefan-Meier-Straße 21, 79104 Freiburg im Breisgau, Germany; 3 Université de Haute Alsace, CNRS, IS2M UMR 7361, 68100 Mulhouse, France; 4 Strasbourg University, 4 Rue Blaise Pascal, 67000 Strasbourg, France

**Keywords:** wood-based biopolymers, lignin, cellulose derivatives, 3D printing, direct ink writing, cationic initiation, radical initiation, photo-crosslinking, Taguchi design

## Abstract

Polymer blends containing up to 70% organosolv lignin content and lyotropic cellulose derivatives have been established as “lignin inks” for direct ink writing of fully biobased 3D parts. However, a fast-crosslinking mechanism is needed to improve throughput and design space. In this paper, UV-photocrosslinkable organosolv lignin/hydroxypropyl cellulose inks are formulated through doping with common photocrosslinkers. The most potent photocrosslinkers for neat hydroxypropyl cellulose, lignin and their blends are determined through a series of DOEs. Hydroxypropyl cellulose is significantly more amenable to photocrosslinking than organosolv lignin. The optimal photocrosslinkable ink formulations are printable and exhibit up to 70% gel content, although thermal post-curing remains essential. Chemical, thermal, and mechanical investigations of the photocrosslinked 3D parts evidence efficient crosslinking of HPC through its hydroxyl groups, while lignin appears internally plasticized and/or degraded during inefficient photocrosslinking. Despite this, photocrosslinkable inks exhibit improved tensile properties, shape flexibility, and fidelity. The heterogeneous crosslinking and residual creep highlight the need to further activate lignin for homogeneous photocrosslinking in order to fully exploit the potential of lignin inks in DIW.

## 1. Introduction

The utilization of renewable and humus-returning wood-based polymers in lieu of petroleum-derived polymers is highly desirable to circumvent the multiple environmental issues associated with synthetic plastics [1,2]. Among the structural biopolymers found in wood, cellulose and lignin complement each other by providing properties in tension and in compression, respectively [3,4,5,6,7,8]. Their combination in bio-based materials presents a promising avenue for the development of high-performance materials capable of withstanding different loadings. When attempting to develop and process blends based on lignin and cellulose, additive manufacturing appears particularly attractive. It offers a more sustainable production process than traditional subtractive technologies owing to its material efficiency and large design space, thereby reducing process energy and material waste [9,10].

Over the past decade, several research groups have thus turned to additive manufacturing for processing wood-based polymers with varying degrees of success [11,12,13]. In most cases, wood-based polymers are combined with synthetic polymers [14,15,16]. However, some research groups have relied exclusively on wood polymers for 3D printing [17,18]. Inspired by the woody cell wall morphogenesis, our research group has also explored the potential of Direct Ink Writing (DIW) blend solutions of lyotropic cellulosic derivatives and organosolv lignin, thereafter coined lignin inks due to their high lignin content of up to 70%. In addition to being fully renewable, these blends are particularly well-suited for DIW because of two key properties: (i) the strong shear-thinning behavior of the liquid crystalline cellulose derivatives, which imparts processability at high shear rates, and (ii) the cementing and solidifying effect of the amorphous lignin at zero-shear rate once the blend is deposited on the printing platform [19,20]. It has indeed been shown that the composition of these blends can be specifically tuned to the required shear thinning and viscoelastic properties for DIW [21,22]. This blending strategy has been validated, and process parameters have been optimized [23,24] for lyotropic solutions of organosolv lignin (OSL) with various cellulose derivatives [25,26]. However, the slow evaporation-driven solidification process and the need for a post-curing step have so far limited the design space and production rates of the parts [19,20,21,22,23]. There is therefore a need to develop faster solidification processes *in situ* for these fully bio-based lignin inks, viz. on the printing platform.

A promising approach to boost the fluid-to-solid transition of polymers in 3D printing relies on photochemistry [27]. Intermolecular photocrosslinking could indeed be easily photo-induced for the lignin inks by shining light on the printing platform, ideally endowing instantaneous integrity, high shape fidelity and high throughput to the bio-based 3D parts. This requires the design of photocrosslinkable lignin inks formulations that retain printability.

**Scheme 1:**
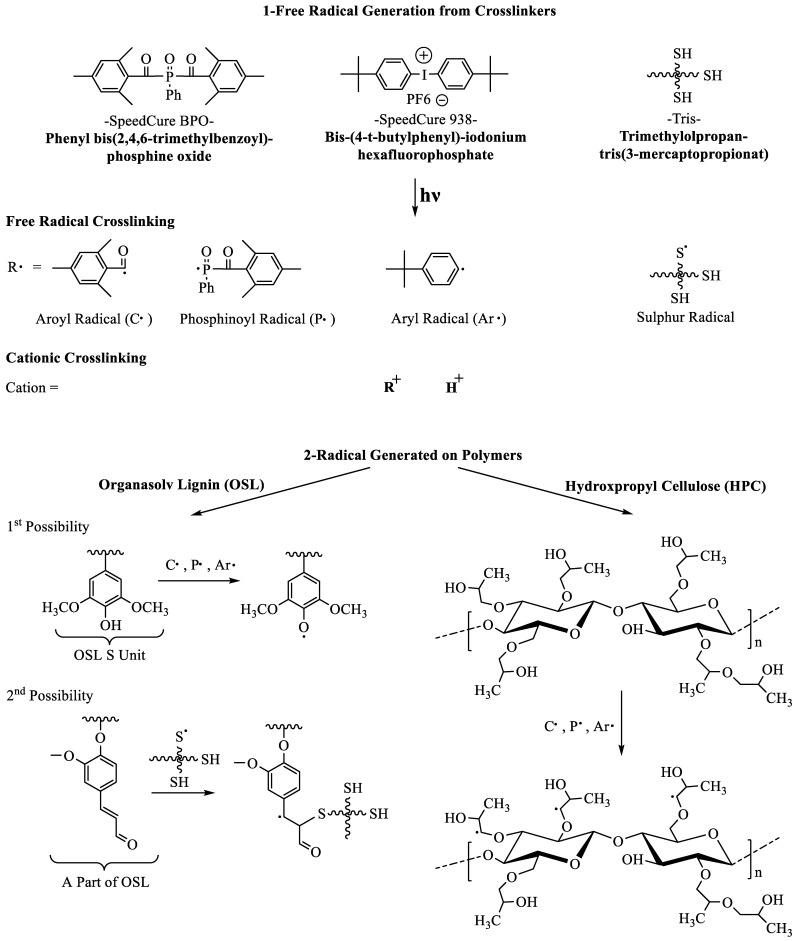
Possible reactive species generated by the photocrosslinking treatment.

While photocrosslinking has been employed on a variety of biopolymers and their blends [28], including lignin, cellulose and its derivatives [29,30,31,32,33], its use in inks composed entirely of wood polymers remains limited [17,18], and to the best of our knowledge, nonexistent for lignin/cellulose derivative LC blends. A thorough study of photo-crosslinking systems for neat lignin, neat cellulosic derivatives, and their lyotropic blends is therefore needed to formulate photocrosslinkable lignin inks for DIW to enable a fast fluid–solid phase transformation. Photocrosslinking can be initiated by free radical and/or cationic photoinitiators [27]. Phenyl bis(2,4,6-trimethylbenzoyl)-phosphine oxide (Speedcure BPO, Arkema) and 1,1,1-Tris-(hydroxymethyl)-propane tris-(3-mercaptopropionate (thereafter labeled Tris in this publication) are common free radical photocrosslinkers, which decompose into various radicalic species upon irradiation at a specific wavelength (Scheme 1). Cationic photoinitiators such as Bis-(4-t-butylphenyl)-iodonium hexafluorophosphate (Speedcure 938, Arkema) also generate free radicals, aryl radicals in this case via reduction of iodonium (Scheme 1). We specifically chose these three crosslinkers for their widespread use, synergetic effects, and capacity to span free-radical and cationic mechanisms. Industrial photocrosslinkable formulations frequently use a combination of both cationic and free-radical photocrosslinkers for enhanced synergistic effects [34]. Cationic photocrosslinkers can indeed prolong the reaction after light exposure by exposure to heat [35]. Additionally, the free-radical crosslinker Tris, a thiol-based compound, can also be thermally cured with or without initiators [36,37,38]. Such synergies have been demonstrated for both petroleum and bio-based systems [39,40]. As pure lignin is a well-known scavenger of free-radicals [41], reaching up to 90% anti-oxidation activity, free-radical routes might not function well for lignin; a cationic system appears a priori more suitable for neat lignin. However, in lignin/cellulosic blends, free-radical crosslinking routes might still be functional as carbohydrates are reported to inhibit lignin’s anti-oxidation activity [41]. In contrast, abundant literature supports the efficacy of free-radical pathways to crosslink cellulose and cellulose derivatives [42].

The overall goal of this work is to develop photocrosslinkable lignin ink formulations based on organosolv lignin (OSL) and hydroxypropyl cellulose (HPC), that are suitable for printing parts through DIW. In the first phase, we report on the potential of common commercial radicalic and cationic photocrosslinkers to photocrosslink neat organosolv lignin, neat hydroxypropyl cellulose and their lyotropic blends. The second phase focuses on assessing the viability of DIW using photocrosslinker-doped OSL/HPC inks as a function of photocrosslinkers and processing parameters. Finally, the physico-chemical and mechanical properties of the resulting printed parts are presented, thereby shedding some light on possible crosslinking mechanisms and their impact on parts’ properties.

## 2. Materials and Methods

### 2.1. Raw Materials

Beech Organosolv lignin (OSL) was generously supplied by the Fraunhofer Center for Chemical-Biotechnological Processes from the Leuna Biorefinery, Germany. Hydroxypropyl cellulose (HPC) with a molecular weight of 100,000 g.mol^−1^ was purchased from Thermo Fisher GmbH (Kandel, Germany). The reader is referred to prior publications for an extensive characterization of these two specific biopolymers [19,20]. Acetone (≥99.5%) and Ethanol (≥99.8%) were purchased from VWR International GmbH (Darmstadt, Germany). The free radical crosslinker Tris (1,1,1-Tris-(hydroxymethyl)-propane tris-(3-mercaptopropionate) (purity ≥ 95.0%), was purchased from Sigma-Aldrich Chemie GmbH (Taufkirchen, Germany). The free radical initiator Speedcure BPO, phenyl bis(2,4,6-trimethylbenzoyl)-phosphine oxide (purity ≥ 96.0%), and the cationic initiator, Speedcure 938, Bis-(4-t-butylphenyl)-Iodonium hexafluorophosphate (purity ≥ 98.0%), were acquired from TCI Deutschland GmbH in Eschborn, Germany. OSL and HPC were oven-dried at 100 °C for 3 h and stored in a desiccator containing Silica gel orange (particle size: 2–5 mm) with an indicator from Carl Roth GmbH, (Karlsruhe, Germany) for an additional 24 h to ensure thorough drying. Moisture content was not specifically determined.

### 2.2. Methods

**Figure 1:**
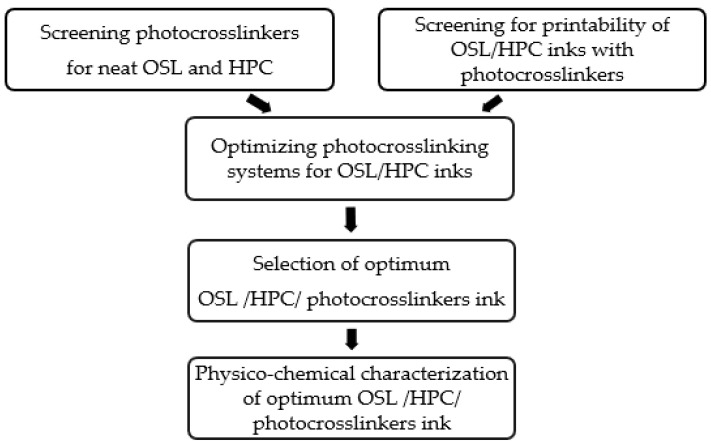
Experimental steps taken to optimize formulations of OSL/HPC inks and photocrosslinking in DIW the inks.

Preliminary investigations were conducted to assess (i) the crosslink-ability under 100% UV light at 395 nm of neat OSL and neat HPC with the selected photocrosslinkers and (ii) the printability of photocrosslinker-doped OSL/HPC inks (Figure 1). Subsequently, an experimental plan was implemented to study the impact of ink formulations and photo crosslinking process parameters on the efficacy of *in situ* photocrosslinking for various lignin/HPC blends. 3D parts printed from the most potent inks were then characterized for physico-chemical properties, morphology, print quality and mechanical performance.

#### 2.2.1. Screening the Potential of 3 Common Photocrosslinkers for Neat Lignin and Neat HPC

A factorial experimental plan was designed to assess the impact of the 3 commercial photocrosslinkers in different loadings as well as their interactions on crosslinking efficacy of the neat OSL and HPC polymers (Table 1).

Each neat biopolymer was dissolved in 70/30 *v/v* acetone/ethanol to reach a 40% solid content (excluding photocrosslinker mass). Note that for best solubility, the crosslinkers were first dissolved in the binary solvent after which the dried polymer was added. Following gentle stirring, the solutions were sealed and stored in the dark at room temperature for 2 days until the UV- photocrosslinking trials. For HPC, photocrosslinking was performed on 30 × 10 × 0.33 mm^3^ HPC single-layered specimens, printed at 5 mm/s, under a printing pressure of 2.5 to 3 bar and using 4th generation Envisiontec 3D-Bioplotter, Developer Series, (Gladbeck, Germany and 0.41mm conical plastic needles (Nordson EFD, Feldkirchen, Germany). For neat organosolv lignin solutions, which were not amenable to DIW, films were solvent-cast in petri dishes with 0.5 mm diameter. Dried film thickness was measured with a caliper and ranged from 0.32 to 0.36 mm Thereafter, the HPC individual specimens and the lignin films samples were placed 40 cm away from a pencil-shaped UV light device emitting 100% UV light at 395 nm for 30 min (Opsytec Dr. Gröbel GmbH, Ettlingen, Germany). Thereafter, both lignin and HPC samples were post-cured in an oven at 90 °C for 24 h. To minimize potential structural degradation from high temperatures in this complex system, which includes lignin (known for its highly intricate structure) and three distinct crosslinkers, we limited the maximum temperature to 90 °C in our experimental design.

For both HPC and lignin samples, crosslinking degree was assessed by weighting the residual insoluble fraction on an ABT 100-5M analytical balance (KERN & SOHN GmbH, Balingen, Germany) following 24-h immersion at 25 °C in ethanol within teabags, extensive washing with ethanol and 24 h drying at 100 °C. While ethanol fully solubilizes HPC, it only partially solubilizes lignin (ca 50%) and is therefore expected to dissolve all non-crosslinked HPC chains and a large fraction of non-crosslinked lignin fragments. The crosslinked material mass was determined as follows:

% crosslinked mass = 100 × (residual dry mass of treated sample after solubilization − residual dry mass of untreated sample after solubilization)/initial mass of untreated sample

The main effects of factors and their interactions on the crosslinking efficacy were assessed with Minitab (version 19.2020.1) by computing:

$$Mean\;of\;Response=\frac{1}{n}\times \sum_{i=1}^{n}yi$$

where yi is the response for each unique combination of factor levels, ‘n’ the total number of experimental runs, which, in this instance, is 8. For factor A in this experimental design for example, the main effect is:

A = 1/8 × (−y1 + y2 − y3 + y4 − y5 + y6 − y7 + y8)

and the response of factor A at each level is:

A (−1) = (y1 + y3 + y5 + y7)/4

A (+1) = (y2 + y4 + y6 + y8)/4

**Figure 2:**
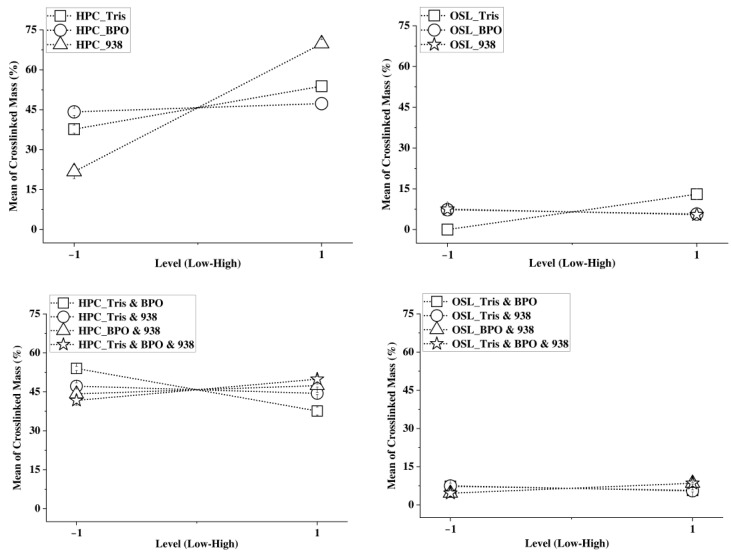
Main effect of each factor (Tris, 938, BPO) (**top**) and factor interaction effect (**bottom**) on the extent of crosslinking in the neat OSL and in the neat HPC.

These computations enable the generation of main effect plots (Figure 2).

#### 2.2.2. Screening for the Printability of OSL/HPC Blends Doped with Crosslinkers

An initial screening process of 3D printability of HPC/OSL formulations with and without crosslinkers was performed according to established protocols [43] using the maximum loading of crosslinker. The ink formulations, prepared with a 50% solid to 50% solvent ratio determined by weight, involved combining OSL and HPC in the same glass container; when introducing nearly maximum amounts of crosslinkers (5% SpeedCure BPO, 5% SpeedCure 938, and 2.5% Tris), they were initially dissolved in a solvent system equivalent to the combined weight of OSL and HPC before being mixed with the polymers. After 2-days the solutions were gently mixed and transferred into 30 cc plastic cartridges (Nordson EFD, Feldkirchen, Germany) for centrifugation under 4500 rpm for 30 min at 25 °C. The formulations were then transferred to plastic conical needle tips (Nordson EFD, Feldkirchen, Germany) of 0.41 mm diameter and extruded manually to observe the quality of fiber formation and layer stacking. The mixtures were also tested for viscosity recovery on an MCR 301 rheometer (Anton Paar, Graz, Austria), employing a cone/plate geometry (25 mm diameter, 2° cone, 0.05 mm truncation), and operating under room conditions. Samples were loaded on the rheometer fixture, sealed with a Krytox GPL 105 (Chemours, Wilmington, DE, USA) mineral oil to prevent evaporation. Rotational shear–viscosity measurements in flow mode were performed over a range of shear rates, spanning from 0.1 to 100 s^−1^. Subsequently, shear rates of 0.01 s^−1^ for 200 s, 895 s^−1^ for 100 s, and 0.01 s^−1^ for 200 s were applied to evaluate the recovery behavior [43].

#### 2.2.3. Optimizing Photo-Crosslinking Systems for DIW of OSL/HPC Inks

A screening Taguchi experimental plan was designed to assess the impact of crosslinker choice and curing parameters on the crosslinking degree of the ink formulations. Namely, an L12(2^7) Taguchi screening plan considering all 3 photo crosslinkers and the UV light intensity and exposure time, post-curing time and temperature as factors at two levels was conducted in triplicates (Table 2).

3D printing, specimen preparation and photocrosslinking were performed in triplicates as previously described (Section 2.2.1 and Section 2.2.2, respectively). Herehowever, the specimens were post-cured at the conditions specified in the L12(2^7) table. The “Larger is better” methodology was used to determine Signal-to-Noise Ratio (S/N) as follows:

$$S/N=-10\times \log_{10}\left(\frac{1}{n}\times \sum_{i=1}^{n}\;\frac{1}{{yi}^{2}}\right)$$

where ‘n’ represents the replication number for each unique combination of factor levels, ‘y’ represents the associated response. For example, for the 1st run:

S/N_1_ = −10 × log_10_ [(y1,1)^2^ + (y1,2)^2^ + (y1,3)^2^)/3]

As illustrated above, the computation of Signal-to-Noise (S/N) ratio values for each experiment run with varying levels of factors is feasible. The calculation methodology provided for the 1st run serves as an illustrative example. Since each experiment was conducted three times, resulting in three y responses for each run, the obtained values were divided by 3.

$$Mean\;of\;Signal\;Noise\;Ratio=\frac{1}{n}\times \sum_{i=1}^{n}SNi$$

SN represents the signal-to-noise ratio for each distinct combination of factor levels, where ‘n’ denotes the total number of experimental runs, which in this case is 12. For example, for factor A, the SN ratio at each level is computed as:

SN_Factor **A**, −1_ = (S/N_1_ + S/N_2_ + S/N_3_ + S/N_4_ + S/N_5_ + S/N_6_)/6

SN_Factor **A**, +1_ = (S/N_7_ + S/N_8_ + S/N_9_ + S/N_10_ + S/N_11_ + S/N_12_)/6

These SN values are used to generate the plot of factor effects.

By utilizing the provided formulation below for each factor, we ranked all parameters, with higher values indicating greater importance:

$$\left|{SN}_{FactorX,-1}-{SN}_{FactorX,+1}\right|$$

This particular study revealed the formulation that crosslinked to the highest extent, which were then considered for further characterization study.

#### 2.2.4. Unravelling the Chemistry and Properties of the Optimized Photo-Crosslinked Ink

Only one formulation was considered in this study to shed some light on the nature of photocrosslinking and its impact on the part properties. To that end an optimally crosslinked sample (50% OSL/50% HPC + 5% SpeedCure BPO, 5% SpeedCure 938, and 2.5% Tris) was compared to a control printed sample (50% OSL/50% HPC) and to neat OSL and HPC. Samples were maintained in a desiccator under dry atmosphere with 2–5 mm Silica gel orange (Carl Roth GmbH, Karlsruhe, Germany) for at least 24-h prior to any characterization.

##### Fourier Transform Infrared Spectroscopy (FTIR)

Spectral data were acquired on triplicate samples using an FTIR spectrometer 65 (PerkinElmer, Shelton, CT, USA) in the attenuated total reflection (ATR) mode, utilizing a ZnSe crystal. 32 scans were collected within the wavelength range of 650 to 4000 cm^−1^ at resolution of 4 cm^−1^. Note that for the HPC samples spectral intensity was normalized to the reference β-D-glucopyranose ring -C-O-C bond vibration [44] at 1050 cm^−1^ [45]. 

For lignin samples, intensity normalization occurred by reference to the in plane deformation of aromatic C–H peak at 1050 cm^−1^ [46]. In the OSL/HPC blend, normalization could theoretically be achieved using either the β-D-glucopyranose ring C-O-C stretch of HPC or the in-plane deformation of the aromatic C-H peak of OSL. However, in our system, these peaks overlap around 1050 cm⁻^1^, which is the region we have utilized for normalization in the blend

##### Dynamic Mechanical Analysis (DMA)

Dynamic mechanical analysis (DMA) was carried out on triplicate samples using a DMA 8000 (PerkinElmer, Shelton, CT, USA) in single cantilever mode on powder specimens wrapped into 30 × 14 mm stainless steel material pockets (Product No. N5330323; Perkin-Elmer, Waltham, MA, USA). Temperature scans were performed at 1 Hz from 25 °C to 200 °C at 2.0 °C/min, using a static force of 2 N and a force multiplier of 1.5. Additionally, a strain of 0.005 mm was maintained throughout the experiment.

##### Microtensile Testing

Micromechanical testing was conducted on five replicate specimens on an Inspection Mini 100N testing machine (Hegewald & Peschke Meß- und Prüftechnik GmbH, Nossen, Germany) operating at 0.4 mm/min in compliance with DIN EN ISO 527-1 standards [47]. Young’s modulus, ultimate tensile strength and toughness were calculated from stress−strain curves with the OriginPro software, Version 2021b (OriginLab Corporation, Northampton, MA, USA). Non-crosslinked samples, initially printed with dimensions of 70 mm (length) × 7.3 mm (width) × 0.32 mm (thickness), underwent air-drying for 3 days to facilitate basic characterization on stress concentration-free specimens. The sample shape design, along with the software and 3D-printing conditions, was previously specified in Section 2.2.1. Following the 3D-printing process, samples were exposed to UV light, subsequently air-dried for 3 days in a dark environment, and subjected to heat in an oven in the selected experimental conditions.

##### Shape Fidelity and Printing Flexibility

To gauge the effect of crosslinking on printing flexibility and shape fidelity, the highest possible cylinder structure was printed on control and optimally crosslinked samples, as determined from visible printing errors and the need for intervention during the printing process. Shape fidelity was further evaluated by visually comparing the highest points along the *y*-axis dimension after printing:

$$∆Y\%=\frac{\left|Y_{printed}-Y_{theorical}\right|}{Y_{printed}}\times 100$$

where Ytheoretical represents the *y*-axis dimension as designed in the Perfactory software (Version 3.2.3594.1909) and Yprinted denotes the measured dimension in the freshly printed part. Furthermore, shape stability was also observed visually after one week of storage at ambient conditions.

##### Thermogravimetric Analysis (TGA)

Thermogravimetric analysis was performed using a Pyris 1 TGA (PerkinElmer, Shelton, CT, USA) under a nitrogen (N_2_) atmosphere, ramping from 25 to 900 °C at a rate of 10 °C min^−1^. Each sample had an approximate weight of 5 mg, and the analysis was conducted three times for each individual sample.

## 3. Results and Discussion

### 3.1. Efficacy of Selected Photo Crosslinkers to Reticulate Neat OSL and Neat HPC

The first preliminary study aimed to identify the nature and combinations of photocrosslinkers that most efficiently crosslinked neat lignin and neat HPC.

Table 3 shows that HPC is much more prone to photocrosslinking than lignin, since the insoluble % gain ranges from 20% to 78% for HPC and only 8 to 20% for lignin.

The main effect plot enables a finer understanding of the impact of each factor and their interaction on the crosslinkability of each neat polymer (Figure 2). For HPC, the most efficient crosslinker is SpeedCure 938, followed by Tris, while BPO has practically no effect in itself. Binary and ternary interactions are also revealed, whereby the Tris/BPO interactions is by far the most significant. Overall, it is clear that HPC very readily crosslinks under the action of photocrosslinkers and in particular to the free cationic photoiniator SpeedCure 938.

OSL is less prone to photocrosslinking than HPC and the photocrosslinkers have much smaller practical effects on lignin than on HPC. This is no surprise considering the well-established antioxidant activity of lignin, which for pure organosolv lignin has been reported as high as 70% [41]. Within this limited potential for lignin, Tris—a light- and heat-induced radical crosslinker—best induces lignin crosslinking compared to the two other systems. While higher amounts of Tris are favorable to lignin crosslinking, a smaller amount of the two other crosslinkers proves advantageous to photocrosslinking of lignin. As for HPC, binary and tertiary interactions are observed but their practical significance is small (Figure 2).

From the separate optimization studies on HPC and lignin photocrosslinking, it was found that HPC could achieve substantial gelation (max 80%), especially when Speedcure 938 was included. In contrast, lignin can only minimally gel (max 20%) and this occurs only when Tris is used. Consequently, both Speedcure 938 and Tris are in principle favorable to crosslink HPC and lignin components, respectively, in multiphase lignin/HPC formulations. Combining these two photocrosslinkers appears favorable for each component due to the binary interactions. While BPO may not be individually impactful, its contribution to crosslinkability in both neat components is significant through its interaction with Tris. These findings prompt the consideration of combining all three photocrosslinkers in the design of photocrosslinkable lignin/HPC inks.

### 3.2. Screening Study of Printability of OSL/HPC Ink Formulation with Various Photocrosslinkers

While multiple OSL/HPC formulations have been found to be amenable to DIW, the addition of photocrosslinkers in the ink may alter its overall viscoelastic properties and impede its printability. To assess the printability of photocrosslinker-loaded lignin/HPC inks, the Paxton procedure is useful as it enables a rapid screening of printability [43]. This protocol was thus conducted on the 50% OSL/50%HPC formulation loaded with the maximum of each photocrosslinker as the most extreme scenario, viz. 5% BPO + 5% SpeedCure 938 and 2.5% Tris. The screening reveals favorable fiber formation and stacked layers in the ink loaded with photocrosslinker, which behaves similarly to the control ink (Table 4).

**Figure 3:**
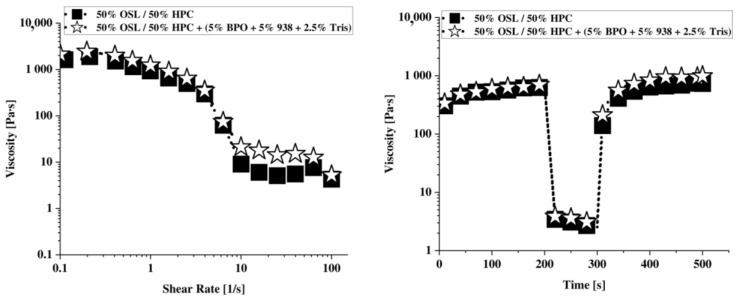
Shear–viscosity (**left**) and viscosity recovery (**right**) for the 50:50 OSL: HPC control ink and the ink of same polymeric composition and supplemented with 5%BPO, 5% P38 and 2.5% Tris photoinitiators.

The shear thinning and the viscosity recoveries are further evidenced in the ink formulation containing the photocrosslinkers (Figure 3). Doping the ink with photo crosslinkers does not change the shear-rate dependency of viscosity notably. The shear recovery behavior of the ink loaded with photocrosslinkers is also sensibly the same as that of the control ink (Figure 3).

Overall, this supports the hypothesis that doping the OSL/HPC inks with photocrosslinkers does not prevent the rheological behavior needed for DIW. The next step thus consisted in optimizing the photocrosslinker systemin the OSL/HPC inks.

### 3.3. Optimizing the Photo-Crosslinking System in the OSL/HPC Inks

In optimizing the photocrosslinking system in the OSL/HPC ink, photocrosslinker nature, amounts and process parameters were all tested in a L2^7 Taguchi experimental plan. Numerous formulations displayed a significant proportion of insoluble mass, surpassing 60%, with formulations 4, 7, 8, and 12 performing particularly well (Table 5).

**Figure 4:**
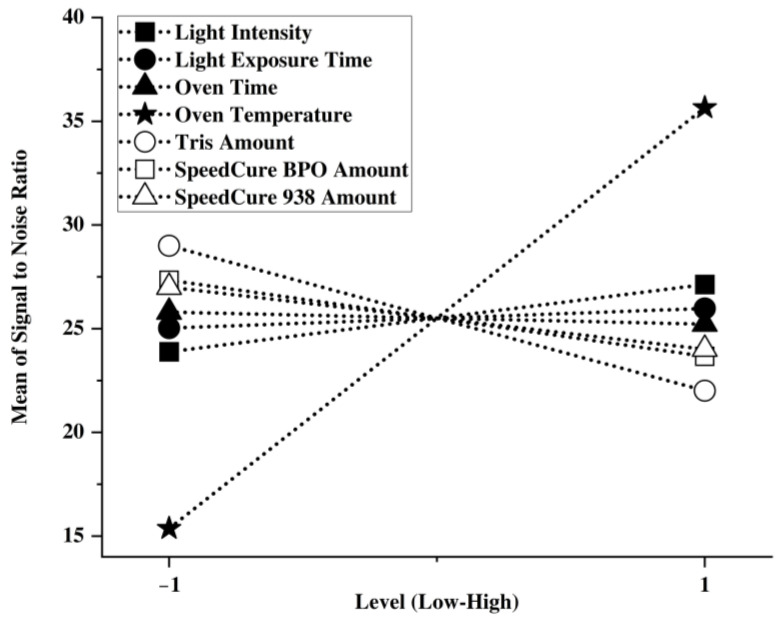
Main effects plot illustrating the individual impact of photocrosslinker and processing factors on crosslinking efficiency in an OSL/HPC ink.

The main factor plots reveal two major effects (Figure 4).

**Figure 5:**
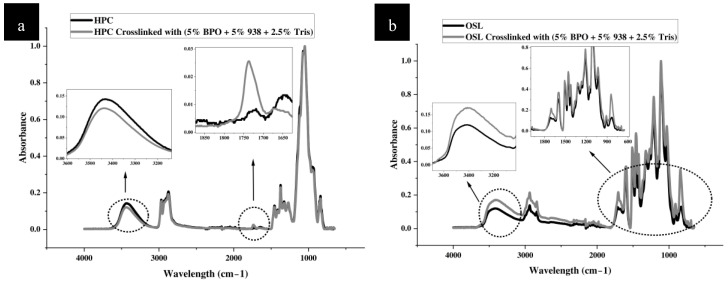

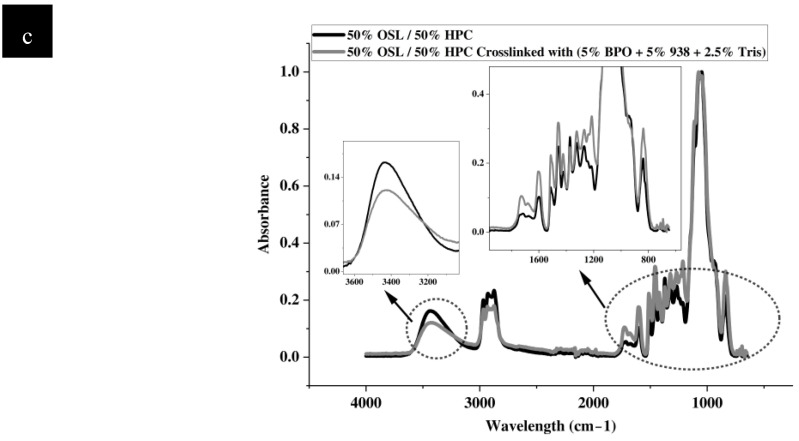
FTIR spectra of control and photocrosslinked (formulation 8) parts, (**a**) HPC, (**b**) OSL, (**c**) OSL/HPC blend. Insets represent a blow up of the fingerprint region and d) OH-stretching vibration in the respective samples.

Firstly, it is evident that oven temperature from the post-cure test is by far the most significant and practical factor for photocrosslinking the ink. In contrast, UV intensity and time have much less impact. This suggests that, in the OSL/HPC systems tested, crosslinking occurs predominantly in the post-cure step (dark curing) through a heat-activated process rather than a UV-activated one. Indeed, literature shows that under heat: (i) existing cationic species, such as those initiated by light from Speedcure 938 become more active [35] and (ii) some free radical photocrosslinkers such as Tris generate additional radicals [36,37,38]. Secondly, photocrosslinkers that have a slight effect on photocrosslinking degree are most effective in smaller amounts (Figure 5). As photoinitiators, excessive amounts would induce a high density of free radicals and rapid termination. Among all three photoiniators, Tris had the most significant influence on crosslinking in the OSL/HPC ink (Figure 5), while Speedcure 939 and BPO had minor influences. To further unravel the photocrosslinking mechanism and its implication on mechanical properties, Formulation 8, which exhibited a high insoluble mass (65.2%), was used for further physico-chemical characterization of the printed part.

### 3.4. Characterization of the Physico-Chemical Properties of Printed Samples in the Optimum OSL/HPC/Photocrosslinker Ink

#### 3.4.1. Insight on the Chemistry of Photocrosslinking

The initial investigation focused on the chemistry of crosslinking via vibrational Infrared spectroscopy (Figure 5). In this analysis, parts printed from 50/50 *w*/*w* % OSL/HPC ink were used as controls for the corresponding photocrosslinked part according to formulation 8. Note that formulation 8 comprises all three photoiniators, notably Tris (2.5%), BPO and Speedcure 938 (5% each), and was subjected to the highest heat and UV intensity during curing. For the control ink, the process parameters were kept identical to those used in the photocrosslinked parts. Characterization was performed before and after the light/heat crosslinking treatment.

Neat HPC notably presents a characteristic broad asymmetric OH stretching vibration with its max at 3433 cm^−1^ and the fingerprint region [48,49] (Figure 5). Upon subjecting HPC to the photocrosslinking treatment, two main changes appear: (i) The OH stretching region exhibits a 4 cm^−1^ shift towards a higher wavelength, accompanied by an approximate 20% reduction in intensity (Table 6), and (ii) a strong carbonyl peak at around 1720 cm^−1^ emerges. These changes indicate that OH groups were altered during the photocrosslinking step. Perhaps crosslinking occurred through the HPC hydroxyl groups and/or oxidation products were generated, albeit the carbonyl signal might simply stem from the presence of Tris (Figure S1).

Following the photocrosslinking treatment, the OSL broad and largely asymmetric OH stretching vibration at 3406 cm^−1^, also undergoes a 5 cm^−1^ shift (to higher wavelengths Figure 5). An apparent increase in OH signal intensity is likely not significant in light of high standard deviations for this measure (Table 6). Simultaneously, a signal increase around 1600 cm^−1^, corresponding to aryl ring stretching, is observed, alongside a shoulder near 880 cm^−1^, indicative of out-of-plane C–H deformation within the aromatic ring. Additionally, we note alterations in the 770–700 cm^−1^ region corresponding to skeletal deformations of aromatic rings, substituent side groups, and side chains. As for HPC, a carbonyl vibration appears at 1720 cm^−1.^ Upon subjecting lignin to the photocrosslinking treatment. Both aromatic and carbonyl vibrations may again simply reflect the presence of the aromatic and carbonyl-bearing photocrosslinkers (Figure S1); they may also reflect oxidation and modifications within lignin aromatic structure. In all cases, it is clear that for both neat HPC and OSL, the OH functionalities are primarily affected.

The same bond vibrations appear affected by the photocrosslinking treatment in the OSL/HPC blends. Specifically, the OH stretching region exhibits a shift of about 5 cm^−1^ towards lower wavelengths and, as for HPC, a significant reduction in intensity by approximately 30%. Surprisingly, the shift is in opposite direction as that observed for the neat polymers while asymmetry sharpens resulting in an OH-stretching band distribution resembling that of neat lignin. Perhaps, OH groups in HPC are more affected viz. consumed during the photocrosslinking treatment, leaving a residual OH signal that approaches that of neat lignin itself, both in its maximum wavelength and in its distribution.

**Figure 6:**
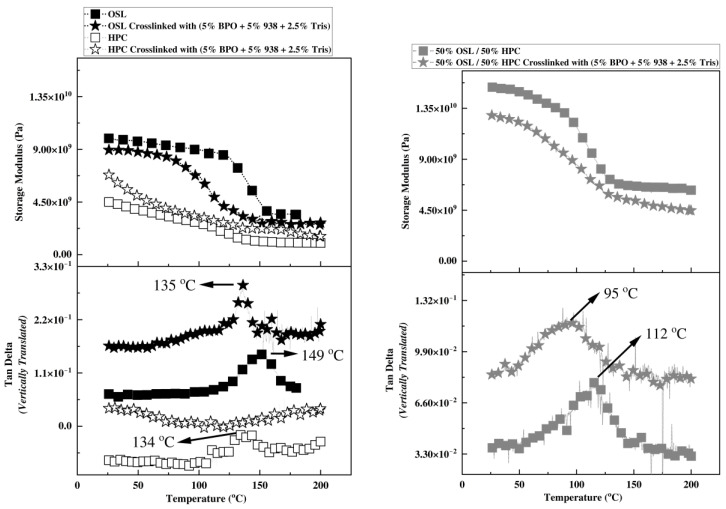
DMAThermograms obtained at 1 Hz and 2 °C/min for isolated polymers (**left**) and OSL/HPC blend (**right**) before and after crosslinking.

Although we observed a clear decrease in OH content after photocrosslinking treatment, O-H bonds are typically not directly involved in free-radical mechanisms, as their bond dissociation energy is too high. However, their environment can be altered by the crosslinking process, such as a shift from secondary to tertiary alcohols (Scheme 1). Additionally, the generation of Brønsted acid, due to the presence of iodonium salt, can further influence their environment. The direct involvement of OH groups in the crosslinking process can however be envisioned with a cationic mechanism. Indeed, it is plausible that some free radicals on the polymer backbone are oxidized into cationic species by the iodonium salt, allowing OH groups to react with these cationic centers, as reported with free-radical-promoted cationic mechanisms [50]. While the observed FTIR changes (wavenumber and signal intensities) confirm chemical changes induced by the photocrosslinking treatments, proposing a crosslinking mechanism would be elusive from these data. At best, the structure of the free radicals generated by each photocrosslinker and possibly on lignin and cellulose can be inferred from chemical reactivities (Figure 6). 

#### 3.4.2. Viscoelastic Properties of Photocrosslinked Parts

A crosslinking reaction is expected to increase the stiffness and storage modulus of a polymer while reducing its free volume, resulting in a Tg increase. In Figure 6, storage modulus increases expectedly in HPC upon the photocrosslinking treatment, confirming effective stiffening upon crosslinking. Variations in analytical methods, molecular weights, substitution degrees, and other factors have led to significant discrepancies and uncertainties in determining the Tg of HPC in the literature, making its precise identification challenging [51]. One preliminary study reported the Tg of HPC as 124 °C [52]. However, the Ashland company specifies a Tg of 150 °C for HPC in its product catalog [53]. In our study, we observed the Tg of HPC, based on the Tan delta peak in our DMA results, to fall within the range of 135–140 °C. Notably, after photocrosslinking, this Tg peak is no longer detectable. The absence of a detectable Tg in highly crosslinked polymers by DMA suggests that the photochemically treated HPC has achieved a high crosslink density. In contrast, neat OSL exhibits a significant decrease of its storage modulus throughout the temperature range; its Tg also decreases by about 20 °C upon photocrosslinking treatment. This unexpected softening might stem from: (i) internal plasticization induced by incomplete reaction of the crosslinkers, and/or from (ii) degradation via chain cleavage of lignin upon exposure to UV in the presence of free radical/cationic initiators. Indeed, one can envision that the photocrosslinkers bond covalently to lignin through one reactive site only, thereby appending a bulky group to lignin backbone, that would increase its free volume. Tris, in particular, with its functionality of 3, its highly flexible S-C bond [54,55,56], and its low Tg, may only react with lignin through one arm, thereby internally plasticizing lignin rather than crosslinking it. This behavior might be further exacerbated by lignin’s poor reactivity in radical systems due to its antioxidative properties [41]. One can also envision chain scission and degradation of the lignin backbone under the action of UV and free radicals. In wood, cellulose and hemicellulose may absorb only 5–20% of UV light, whereas lignin absorbs approximately 80–95% making it more susceptible to decomposition by UV light [57,58]. Lignin is known to absorb wavelengths in the 295–400 nm range [59] and dissociate moieties containing carbonyl, biphenyl, or ring-conjugated structures [60], even without a catalyst [61]. Lignin is therefore likely to degrade more readily than HPC upon the UV photocrosslinking treatment at 395 nm. Therefore, thermal post-curing appears to be the most effective method compared to alternative approaches, such as prolonged UV exposure in a chamber.

Previous research on a similar lignin inks demonstrated that crosslinking resulted in significantly enhanced mechanical properties. In our system, the dynamic mechanical thermogram of the inks reveals a significant decrease of storage modulus throughout the glassy and rubbery states together with a decrease of the single Tg from 95 °C to 112 °C upon photocrosslinking. The drop in storage modulus and Tg values is reminiscent of the neat lignin’s behavior upon photocrosslinking and might likewise stem from lignin low reactivity and incomplete crosslinking in addition to possible degradation reactions. Also note that similar drop in lignin Tg has been observed when attempting to crosslink a rigid thermosetting oligomeric phenolic resin into wood, and ascribed to incomplete crosslinking and network formation as a result of simple lack of proximity of reactive sites or steric hindrance [62].

#### 3.4.3. Tensile Tests

**Figure 7:**
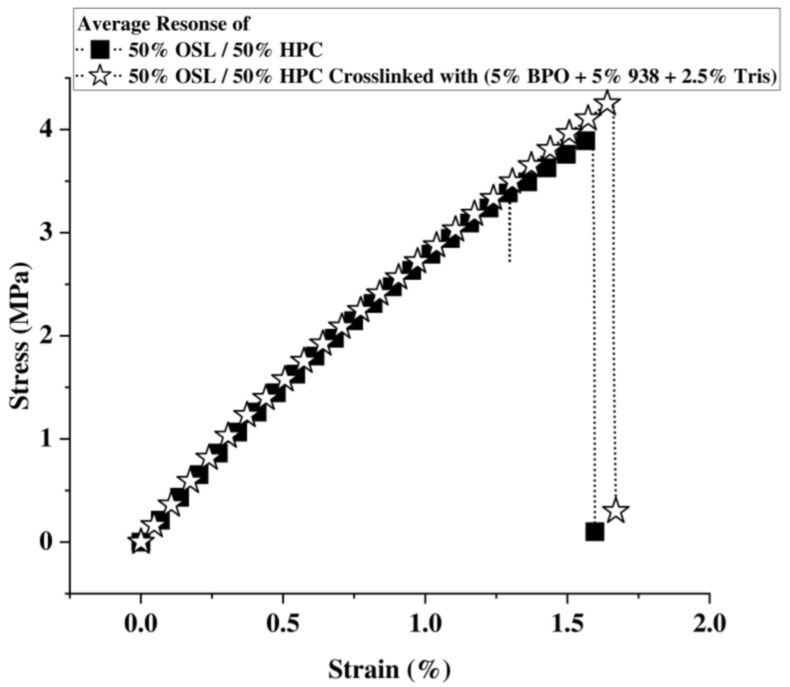
Stress–strain curves of samples printed with 50% HPC and 50% OSL: (**left**) before crosslinking, (**right**) after crosslinking.

After subjecting the ink to the photocrosslinking treatment, the original tensile stress strain behavior as a glassy solid with low plasticity is maintained (Figure 7). Note that the tensile properties of the lignin inks developed in this work appear all significantly lower than those measured in an optimized process with a similar ink but using a distinct solvent system and also testing another curing chemistry. In lignin/HPC inks, noncrosslinked parts already exhibited UTS of 13 MPA and moduli of 400 MPA, well above those measured here [23].

However, a slight stiffening is observed in the linear elastic region, with the Young’s modulus increasing by approximately 20%. Likewise, elongation at break and ultimate strength slightly increase, viz. by circa 10% (Table 7). However, a more substantial enhancement is noted in the toughness, which increases by 25% following the photocrosslinking treatment. The more rubbery and softer state of lignin likely enhances the damping efficiency of the photocrosslinked parts, thereby increasing its toughness. These increases remain surprisingly moderate compared to prior work. Ebers [23] indeed observed 50 % modulus increase up to almost 900 MPa. As discussed in the DMA results, degradation possibly limited crosslinking efficiency for lignin and the flexibility of some photocrosslinkers might also have contributed to the overall moderate change observed.

#### 3.4.4. Shape Fidelity and Stability

To best endow shape fidelity, integrity and stability in 3D parts based on polymer blends, intermolecular crosslinking should not only occur within the bulk of each polymer but also at their interface (Scheme 1). Shape fidelity and stability were thus compared for the lignin inks before and after photocrosslinking treatment.

**Figure 8:**
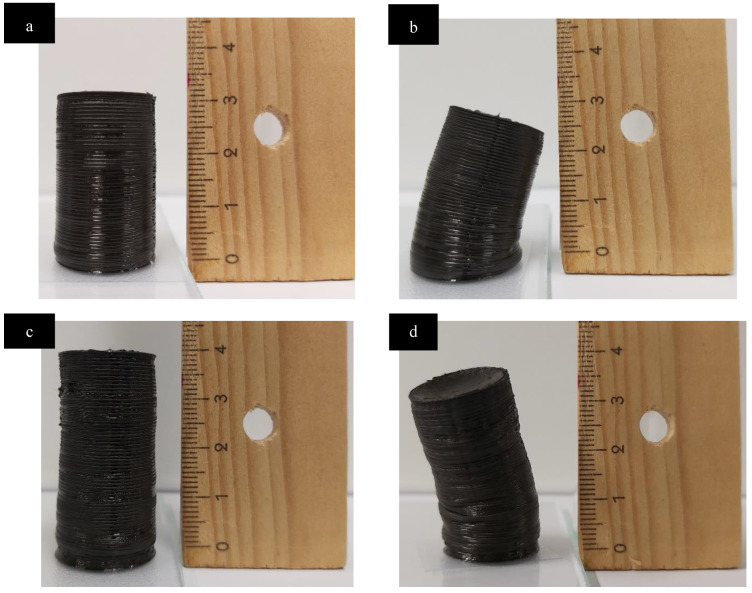
(**a**,**b**): Tallest (100 layers) possible cylinder printed with a Control ink (50% OSL/50% HPC, no crosslinker) in its maximum height immediately post-printing and after 1 week of storage in ambient conditions. (**c**,**d**): Tallest (125 layers) Cylinder printed with Photocrosslinkable Ink (of 50% OSL/50% HPC with 5% BPO + 5% 938 + 2.5% Tris), immediately post-printing and after 1 week.

Note that compared to a previous study on similar lignin inks [23], approximately 40 additional layers could be printed without the need for a cross-linker, possibly as a result of the higher solid content and a different solvent system (Figure 8a,b). The photocrosslinkable formulations subjected to the photocrosslinking treatment augmented the part height by another 25 layers (Figure 8c,d). In addition to enabling the production of taller parts, photocrosslinking augmented shape fidelity from 3 ± 0.23% to 2.4 ± 0.1%. This is significantly better than in similar lignin inks cured thermally in a post-printing treatment, although their objects were much smaller and values of shape fidelities in that prior work may not be comparable with our values [23]. Yet, upon storage at ambient conditions, all the parts including those that were subjected to photocrosslinking, displayed significant creep. This supports the proposition that despite efficient photocrosslinking within the bulk HPC phases, insufficient crosslinking might have occurred in other phases viz. in the lignin phase and possibly at the lignin/HPC interface. This poor stability might not be an intrinsic problem of the ink but rather reflect the incomplete UV irradiation of the samples since the UV light source was shun from one side of the part, leaving little UV exposure for the other side.

The remaining slow solidification and resulting instability of 3D-printed objects indicate that despite some improvement for faster UV curing and enhanced part modulus, research on optimum photocrosslinkable lignin inksremain essential. Further efforts should therefore concentrate on enhancing lignin amenability to photocrosslinking, which might best be conducted by endowing lignin with specific photocrosslinkable moieties such as acrylates. Such efforts will be the subject of a subsequent publication.

## 4. Conclusions

1. Both neat HPC and neat organosolv lignin, when doped with free radical and cationic photocrosslinkers, successfully underwent UV photocrosslinking, although lignin exhibited significantly less gel formation than HPC.
2. Lignin/HPC Inks doped with common photocrosslinkers retained their printability and could be processed with Direct Ink Writing followed by UV photocrosslinking on the printing platform. Yet, a thermal post-cure appeared necessary for more extensive gel formation.
3. An optimum photocrosslinkable lignin/HPC ink, comprising free radical and cationic photocrosslinkers acting synergistically, enabled the printing of significantly taller parts with improved shape fidelity in the y direction, despite a lingering propensity to creep, at least when using the current UV-source set-up, that could not deliver UV light uniformly on the part in printing.
4. FTIR analysis and DMA confirmed heterogeneous photocrosslinking within the multiphase lignin inks, with preferential photocrosslinking occurring in the HPC phase affecting its OH functionalities. In contrast, lignin exhibited a significant softening that could be ascribed to internal plasticization through incompletely reacted photocrosslinkers and/or to lignin degradation.
5. Despite significant improvements in part height, shape fidelity, and tensile properties—particularly in toughness—the photocrosslinked parts remained excessively prone to creep.
6. Further research on lignin modification with photocrosslinkable moieties is needed to develop high-lignin-content, photocrosslinkable lignin/HPC inks.

## Figures and Tables

**Table 1:** 2^3 Full factorial experimental design for determining photo-crosslinkability in neat OSL and neat HPC; Factors A (SpeedCure BPO), B (Tris), C (SpeedCure 938), Response (crosslinked mass, %) with low and high level.

	SpeedCure BPO	Tris	SpeedCure 938	Response
Level −1	0%	2.5%	0%	crosslinked mass, %
Level +1	5%	10%	5%	

**Table 2:** L12(2^7) Taguchi experimental design for optimizing photo-crosslinking of lignin/HPC inks; Factors A (Light Intensity), B (Light Exposure Time), C (SpeedCure BPO), D (Tris), E (SpeedCure 938), F (Oven Time), G (Oven Temperature), Response (crosslinked mass, %) with low and high level, N = 3.

	Light Intensity	Light Exposure Time	SpeedCure BPO	Tris	SpeedCure 938	Oven Time	Oven Temperature	Response
Level −1	10%	30 min	1%	2.5%	1%	6 h	50 °C	crosslinked mass, %
Level +1	100%	120 min	5%	10%	5%	24 h	90 °C	

**Table 3:** Table of responses for the two Full factorial experimental plans including viz. in neat OSL and in neat HPC; Factors A (SpeedCure BPO), B (Tris), C (SpeedCure 938), Response for OSL (% crosslinked mass for OSL), Response for HPC (% crosslinked mass for HPC), N = 3.

Run	A	B	C	AB	AC	BC	ABC	Response for OSL	Response for HPC
1	−1	−1	−1	+1	+1	+1	−1	0	0
2	+1	−1	−1	−1	−1	+1	+1	20.4 ± 0.3	43.5 ± 1.8
3	−1	+1	−1	−1	+1	−1	+1	0	24.5 ± 2.1
4	+1	+1	−1	+1	−1	−1	−1	9.6 ± 1.7	18.9 ± 7.8
5	−1	−1	+1	+1	−1	−1	+1	0	55.8 ± 6.9
6	+1	−1	+1	−1	+1	−1	−1	8.6 ± 0.6	77.3 ± 0.7
7	−1	+1	+1	−1	−1	+1	−1	0	70.4 ± 0.2
8	+1	+1	+1	+1	+1	+1	+1	13.3 ± 0.6	75.5 ± 0.5

**Table 4:** Initial assessment of bioinks: exploration of 50% OSL/50% HPC compositions at 50% solid content using the selected solvent system with and without crosslinking system.

	Without Crosslinker	With Crosslinker (5% BPO + 5% 938 + 2.5% Tris)
Fiber Formation	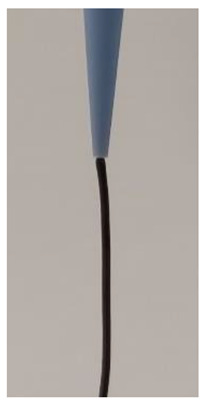	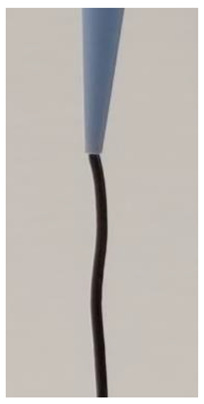
Layer Stacking	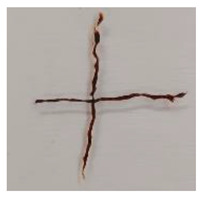	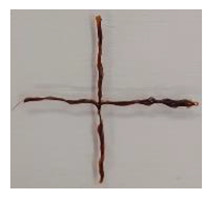

**Table 5:** L12(2^7) Taguchi experimental design for optimizing photo-crosslinking of lignin/HPC inks; Factors A (Light Intensity), B (Light Exposure Time), C (SpeedCure BPO), D (Tris), E (SpeedCure 938), F (Oven Time), G (Oven Temperature), Response (crosslinked mass, %), N = 3.

	Factors and Levels of L2^7 Table							
Run	A	B	C	D	E	F	G	Response
1	−1	−1	−1	−1	−1	−1	−1	21.6 ± 3.3
2	−1	−1	−1	−1	−1	+1	+1	48.9 ± 2
3	−1	−1	+1	+1	+1	−1	−1	0.5 ± 0.2
4	−1	+1	−1	+1	+1	−1	+1	70.7 ± 0.7
5	−1	+1	+1	−1	+1	+1	−1	6.8 ± 4.7
6	−1	+1	+1	+1	−1	+1	+1	53.3 ± 2.5
7	+1	−1	+1	+1	−1	−1	+1	61.5 ± 1.4
8	+1	−1	+1	−1	+1	+1	+1	65.2 ± 0.6
9	+1	−1	−1	+1	+1	+1	−1	14.1 ± 3.6
10	+1	+1	+1	−1	−1	−1	−1	16.1 ± 4.6
11	+1	+1	−1	+1	−1	+1	−1	2.3 ± 0.7
12	+1	+1	−1	−1	+1	−1	+1	67.1 ± 0.5

**Table 6:** Characteristics of FTIR OH stretching vibration (maximum absorbance wavelength and signal area) for isolated polymers and for OSL/HPC inks before and after crosslinking.

Substance	Max. Abs. Wavelength of OH Stretching Vibration (cm^−1^)	Area of OH Stretching Vibration
HPC	3433 ± 2	33.2 ± 3.9
HPC Crosslinked with (5% BPO + 5% 938 + 2.5% Tris)	3437 ± 2	26.4 ± 0.3
OSL	3406 ± 5	28.4 ± 2.7
OSL Crosslinked with (5% BPO + 5% 938 + 2.5% Tris)	3411 ± 2	32.2 ± 6.9
50% OSL/50% HPC	3432 ± 1	38.9 ± 0.8
50% OSL/50% HPC Crosslinked with (5% BPO + 5% 938 + 2.5% Tris)	3427 ± 4	25.5 ± 4.5

**Table 7:** Young’s Modulus, Ultimate Tensile Strength (UTS), Elongation at break and Toughness for OSL/HPC parts before and after crosslinking treatment.

	50% OSL/50% HPC	50% OSL/50% HPC Crosslinked with (5% BPO + 5% 938 + 2.5% Tris)
Modulus (MPa)	316.2 ± 19.9	375.6 ± 35.3
UTS (MPa)	3.7 ± 0.4	4.2 ± 0.5
Elongation (%)	1.5 ± 0.1	1.6 ± 0.3
Toughness (J/m^3^)	3.2 ± 0.6	4 ± 1.4

## Data Availability

Data are contained within the article or Supplementary Material.

## Supplementary Materials

The following supporting information can be downloaded at: https://www.mdpi.com/article/10.3390/polym16202869/s1, Table S1: P values obtained by crosslinking mass percentage results of isolated OSL and HPC, Table S2: Assessment of parameter significance and the corresponding relative effect rankings on the response for 50% OSL/50% HPC crosslinked with (5% BPO + 5% 938 + 2.5% Tris), Table S3: Tg point based on Tan Delta Peaks (oC) from DMA for isolated polymers and OSL/HPC blend before and after crosslinking, Table S4: Summary of TGA results; Figure S1: FTIR spectra of crosslinkers, Figure S2: TGA results for isolated polymers and OSL/HPC blend before and after crosslinking.

## Abbreviations

OSL: organosolv lignin; HPC, hydroxypropyl cellulose; DIW, direct ink writing; SpeedCure BPO or BPO, phenyl bis(2,4,6-trimethylbenzoyl)-phosphine oxide; SpeedCure 938 or 938, bis-(4-t-butylphenyl)-Iodonium hexafluorophosphate; Tris, 1,1,1-Tris-(hydroxymethyl)-propan-tris-(3-mercaptopropionat).
