# Supplementary material for: Towards Photocrosslinkable Lyotropic Blends of Organosolv Lignin and Hydroxypropyl Cellulose for 3D Printing by Direct Ink Writing
Source: Polymers (Basel). 2024 Oct 10;16(20):2869. doi: 10.3390/polym16202869 (PMC11510783; doi:10.3390/polym16202869)
Supplement: Supplementary file 1 [file polymers-16-02869-s001.zip › polymers-3192453-supplementary.pdf]

SUPPORTING INFORMATION

Statically analysis results for crosslinking mass percentage, FTIR spectra of crosslinkers, further thermal characterization results (DMA and TGA)

Table S1. P values obtained by crosslinking mass percentage results of isolated OSL and HPC.

	P-Value-OSL	P-Value-HPC
Tris	1E-14	1.89002E-08
SpeedCure BPO	0.0244736	0.062107385
SpeedCure 938	0.00033328	1E-15
Tris*SpeedCure BPO	0.0244736	1.52302E-08
Tris*SpeedCure 938	0.00033328	0.090960279
SpeedCure BPO*SpeedCure 938	2.4392E-06	0.058606486
Tris*SpeedCure BPO*SpeedCure 938	2.4392E-06	8.3E-05
R-sq	98.27%	98.67%

Table S2. Assessment of parameter significance and the corresponding relative effect rankings on the response for 50% OSL / 50% HPC crosslinked with (5% BPO + 5% 938 + 2.5% Tris)

	**P-Value**	**Rank**
Light Intensity (%)	0.492	4
Light Time (min)	0.898	6
Amount of BPO (%)	0.546	3
Amount of Tris (%)	0.51	2
Amount of 938 (%)	0.556	5
Oven Time (h)	0.219	7
Oven Temperature (C)	0.001	1
R Square	95.91 %	

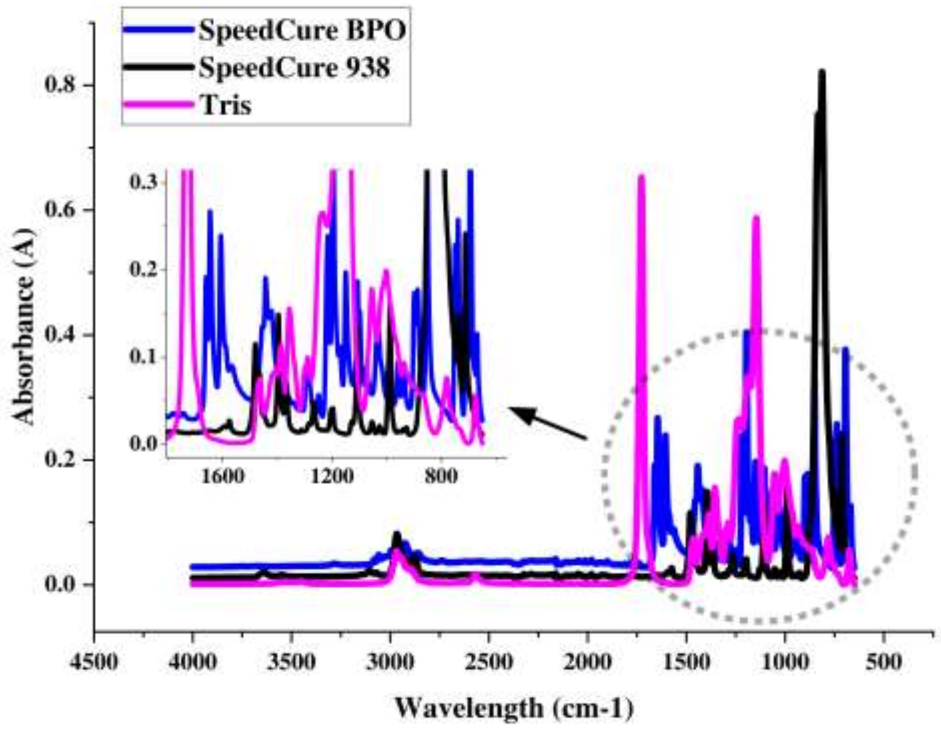

Figure S1. FTIR spectra of crosslinkers

Table S3. Tg point based on Tan Delta Peaks (ºC) from DMA for isolated polymers and OSL/HPC blend before and after crosslinking

Substance	Tg point (ºC)
OSL	149 ± 6
OSL Crosslinked with (5% BPO + 5% 938 + 2.5% Tris)	135 ± 6
HPC	134 ± 3
HPC Crosslinked with (5% BPO + 5% 938 + 2.5% Tris)	No observation
50% OSL / 50% HPC	112 ± 2
50% OSL / 50% HPC Crosslinked with (5% BPO + 5% 938 + 2.5% Tris)	95 ± 8

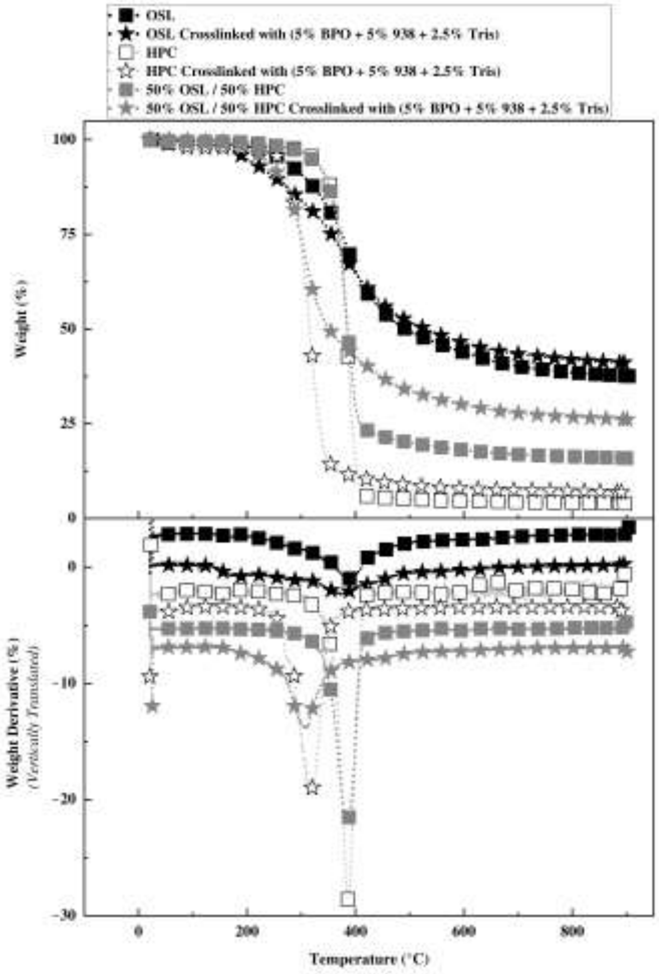

Figure S2. TGA results for isolated polymers and OSL/HPC blend before and after crosslinking

Table S4. Summary of TGA results

	OSL	OSL Crosslinked with (5% BPO + 5% 938 + 2.5% Tris)	HPC	HPC Crosslinked with (5% BPO + 5% 938 + 2.5% Tris)	50% OSL / 50% HPC	50% OSL / 50% HPC Crosslinked with (5% BPO + 5% 938 + 2.5% Tris)
Water Content (%)	1.2 ± 0.5	0.4 ± 0.1	1.6 ± 0.2	1.1 ± 0.3	0.3	0.3 ± 0.2
Tonset (°C)	268.7 ± 88.2	257.3 ± 30.6	354.3 ± 25.5	297.3 ± 2.9	355.7 ± 4.2	205.8 ± 94.4
Tdegredation (°C)	388.7 ± 4.9	376.3 ± 12.1	388 ± 2	317.3 ± 2.1	384.3 ± 3.2	309.3 ± 1
Residual Mass (%)	37.1±0.8	40.2 ± 0.9	4 ± 0.1	6.7 ± 0.3	15.7 ± 0.3	26.1± 0.3
